# Adaptive Gene Amplification As an Intermediate Step in the Expansion of Virus Host Range

**DOI:** 10.1371/journal.ppat.1004002

**Published:** 2014-03-13

**Authors:** Greg Brennan, Jacob O. Kitzman, Stefan Rothenburg, Jay Shendure, Adam P. Geballe

**Affiliations:** 1 Division of Human Biology, Fred Hutchinson Cancer Research Center, Seattle, Washington, United States of America; 2 Department of Genome Sciences, University of Washington, Seattle, Washington, United States of America; 3 Division of Biology, Kansas State University, Manhattan, Kansas, United States of America; 4 Division of Clinical Research, Fred Hutchinson Cancer Research Center, Seattle, Washington, United States of America; 5 Departments of Microbiology and Medicine, University of Washington, Seattle, Washington, United States of America; University of Florida, United States of America

## Abstract

The majority of recently emerging infectious diseases in humans is due to cross-species pathogen transmissions from animals. To establish a productive infection in new host species, viruses must overcome barriers to replication mediated by diverse and rapidly evolving host restriction factors such as protein kinase R (PKR). Many viral antagonists of these restriction factors are species specific. For example, the rhesus cytomegalovirus PKR antagonist, RhTRS1, inhibits PKR in some African green monkey (AGM) cells, but does not inhibit human or rhesus macaque PKR. To model the evolutionary changes necessary for cross-species transmission, we generated a recombinant vaccinia virus that expresses RhTRS1 in a strain that lacks PKR inhibitors E3L and K3L (VVΔEΔK+RhTRS1). Serially passaging VVΔEΔK+RhTRS1 in minimally-permissive AGM cells increased viral replication 10- to 100-fold. Notably, adaptation in these AGM cells also improved virus replication 1000- to 10,000-fold in human and rhesus cells. Genetic analyses including deep sequencing revealed amplification of the *rhtrs1* locus in the adapted viruses. Supplying additional *rhtrs1* in *trans* confirmed that amplification alone was sufficient to improve VVΔEΔK+RhTRS1 replication. Viruses with amplified *rhtrs1* completely blocked AGM PKR, but only partially blocked human PKR, consistent with the replication properties of these viruses in AGM and human cells. Finally, in contrast to AGM-adapted viruses, which could be serially propagated in human cells, VVΔEΔK+RhTRS1 yielded no progeny virus after only three passages in human cells. Thus, *rhtrs1* amplification in a minimally permissive intermediate host was a necessary step, enabling expansion of the virus range to previously nonpermissive hosts. These data support the hypothesis that amplification of a weak viral antagonist may be a general evolutionary mechanism to permit replication in otherwise resistant host species, providing a molecular foothold that could enable further adaptations necessary for efficient replication in the new host.

## Author Summary

The spread of microbes from animals to humans has been responsible for most recently emerging human infectious diseases, including AIDS, bird flu, and SARS. Therefore, understanding the evolutionary and molecular mechanisms underlying cross-species transmission is of critical importance for public health. After entering a new host cell, the success of a virus depends on its ability to overcome antiviral factors in the cell, such as protein kinase R (PKR). To investigate the process of virus transmission between species, we employed a recombinant vaccinia virus (VVΔEΔK+RhTRS1) expressing the rhesus cytomegalovirus PKR antagonist RhTRS1. This protein inhibits some African green monkey (AGM) PKRs; however, it does not inhibit human or rhesus variants of PKR. Serial passaging VVΔEΔK+RhTRS1 in RhTRS1-resistant AGM cells resulted in *rhtrs1* duplication in the viral genome, which improved VVΔEΔK+RhTRS1 replication in AGM cells. Remarkably, *rhtrs1* duplication also enhanced virus replication in human and rhesus cells. In contrast, passage of VVΔEΔK+RhTRS1 in human cells, without prior adaptation in AGM cells, did not improve VVΔEΔK+RhTRS1 replication. These results support the hypothesis that amplification of a weak viral antagonist of a host defense protein in one species may enable cross-species transmission into new hosts that are nonpermissive to the initial virus.

## Introduction

There are at least 868 described zoonotic microbial pathogens, 33% of which are capable of human to human transmission [1]. Recent viral zoonoses have led to some of the most devastating and medically relevant outbreaks in modern history, including SARS coronavirus, pandemic influenza, and HIV/AIDS, highlighting the urgent need to understand how viruses adapt to infect new species. At a population level, factors influencing the transmission of zoonotic pathogens to humans include increasing population density, greater contact with wildlife, increased travel, and poor public health infrastructure [2], [3]. However, these factors only allow the microbe increased access to new hosts; they do not directly enable it to adapt to and replicate in the new species. Intermediate hosts, animals that are not the natural host of a virus but are still permissive or semi-permissive for viral replication, play a critical role in cross-species transmission. These hosts can facilitate increased contact between a virus and a new host, and drive adaptive changes that may improve virus replication (Reviewed in [4]). For example, spill-over of Nipah virus from fruit bats into pigs, the intermediate host, increased human exposure to the virus and resulted in eventual human outbreaks in Malaysia [5], [6]. In another example, lentiviral adaptation through intermediate chimpanzee hosts led to both increased contact with humans, and adaptive genetic changes permitting the virus to inhibit the human versions of several host restriction factors (Reviewed in [7]).

At a molecular level, the initial success of a virus after entry into a new host cell depends on its ability to overcome cellular host restriction factors. A subset of these proteins inhibits specific virus families, such as the restriction of retroviruses mediated by TRIM5α [8]. However, other restriction factors, including protein kinase R (PKR), block the replication of multiple virus families. PKR is activated by binding to double-stranded RNA (dsRNA), a common byproduct of both RNA and DNA virus replication, followed by dimerization and autophosphorylation. Activated PKR then phosphorylates the α-subunit of eukaryotic initiation factor 2 (eIF2α), ultimately arresting translation initiation [9]. In response to the broad and potent barrier to viral infection imposed by PKR, most virus families have evolved at least one mechanism to inhibit the PKR pathway [10].

This conflict between host restriction factors and their viral antagonists results in an “arms race” leading to rapid evolution of both sets of genes [11]. The extraordinarily high rate of positive selection (dN/dS) among primate PKR genes reflects the evolutionary pressure on PKR to evade virus antagonists [12], [13]. To productively infect new host species, virus antagonists must rapidly adapt to escape these differences in PKR. In the current study, we modeled the process of viral adaptation through an intermediate, minimally permissive host. We experimentally evolved a recombinant vaccinia virus expressing the rhesus cytomegalovirus PKR antagonist *rhtrs1* in African green monkey cells expressing RhTRS1-resistant PKR. We demonstrate that amplification of the exogenous *rhtrs1* locus was an early adaption that is sufficient to rescue virus replication in minimally-permissive AGM cells. This amplification of the *rhtrs1* locus was also sufficient to expand the species tropism of these viruses, enabling them to infect both human and rhesus cells substantially better than the initial virus. Importantly, *rhtrs1* amplification did not occur when the initial virus was passaged through human fibroblasts, suggesting that amplification in AGM cells was a necessary intermediate step to expand the virus host range. Gene amplification is a universal mechanism of rapid adaptation occurring in eukaryotes [14], [15], prokaryotes [16], and viruses [17], [18], enabling diverse adaptations including evasion of host restriction factors (Reviewed in [19]). Our results suggest that gene amplification in an intermediate host may be a risk factor for broad cross-species transmission independent of other adaptive events.

## Results

### Variable inhibition of African green monkey PKR by RhCMV TRS1

**Figure 1:**
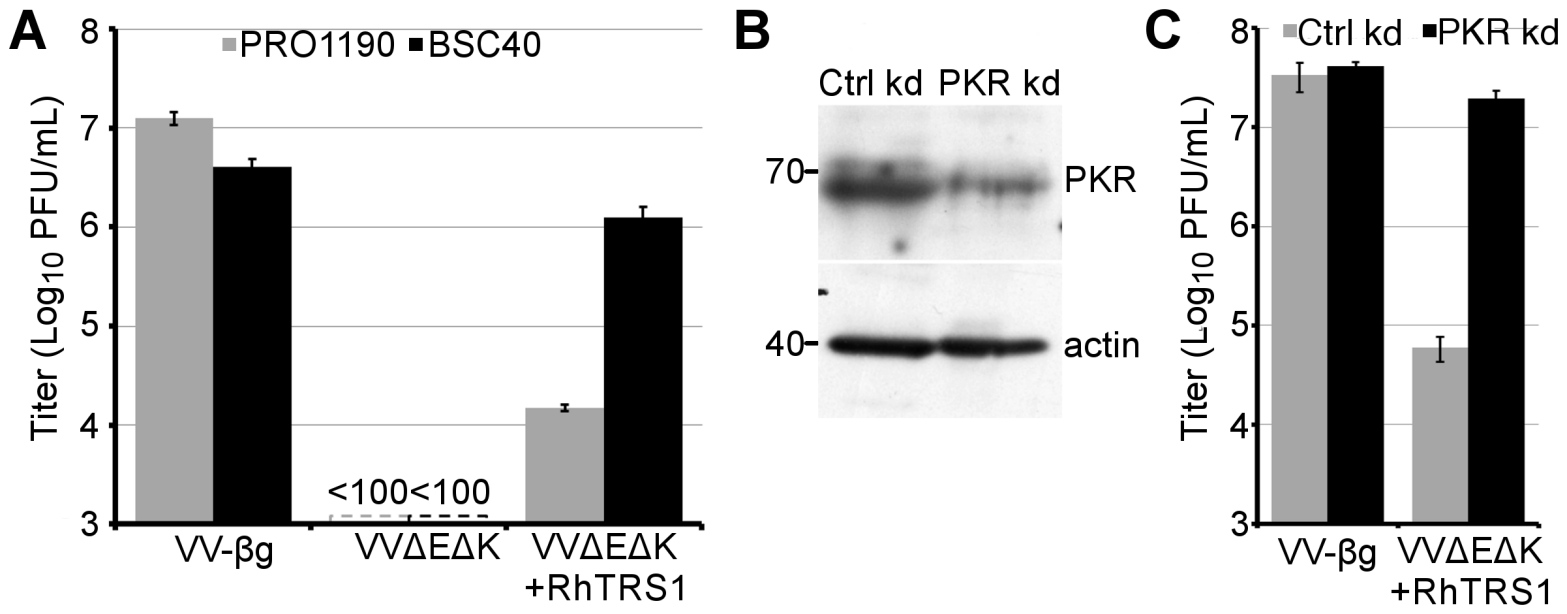
PKR inhibits VVΔEΔK+RhTRS1 replication in PRO1190 cells. (A) Titers of the indicated viruses produced 48 hpi (MOI = 0.1) of PRO1190 (gray bars) or BSC40 cells (black bars), measured using HFF+TRS1. (B) Immunoblot of lysates of PRO1190 stably transduced with lentiviral vectors expressing control (left) or PKR-specific (right) shRNAs, probed with anti-PKR or anti-actin antibodies. (C) Titers of the indicated viruses 48 hpi (MOI = 0.1) in either PRO1190+ctrl shRNA (grey bars) or PRO1190+PKR shRNA (black bars), measured using BSC40 cells. Results are represented as means +1 STD.

The rhesus cytomegalovirus (RhCMV) PKR inhibitor TRS1 (RhTRS1) can block PKR activation and rescue replication of a vaccinia virus mutant lacking the PKR inhibitor E3L (VVΔE3L+RhTRS1) in several African green monkey (*Chlorocebus aethiops*, AGM) cell lines [20]. However, we discovered that a recombinant vaccinia virus expressing RhTRS1 and lacking both of the known vaccinia PKR antagonists, E3L and K3L, (VVΔEΔK+RhTRS1) produced 100 to 1000-fold less virus in AGM-derived PRO1190 cells relative to VV-βg (which contains both E3L and K3L) although it replicated almost as efficiently as VV-βg in AGM-derived BSC40 cells (Fig. 1A). Thus, RhTRS1 varies in its ability to support VVΔEΔK replication in different AGM cells.

This VVΔEΔK+RhTRS1 replication defect in PRO1190 cells may be due to incomplete inhibition of PKR in these cells by RhTRS1. To test this hypothesis, we generated PRO1190 cells stably expressing either a PKR-specific shRNA (PRO1190-PKR kd), which resulted in a 56% reduction of PKR expression, or a scrambled control shRNA (PRO1190-ctrl kd) (Fig. 1B). Similarly, PKR-specific RT-qPCR demonstrated a 60% reduction in PKR mRNA from PRO1190-PKR kd cells relative to PRO1190-ctrl kd cells (2578 copies or 6503 copies PKR/ng total RNA respectively) but little difference between PRO1190 and PRO1190-ctrl kd cell PKR levels (Fig. S1). In PRO1190-PKR kd cells, VVΔEΔK+RhTRS1 replication was almost completely rescued to VV-βg levels (Fig. 1C). We detected a similar increase in VVΔEΔK+RhTRS1 replication after transiently transfecting PRO1190 cells with a siRNA specific for PKR, but not a control siRNA (data not shown). Sequence analysis of PRO1190 PKR identified three non-synonymous mutations relative to a previously reported AGM PKR (GenBank # EU733254) that is sensitive to RhTRS1 (Fig. S2, [20]). Interestingly, one of these single nucleotide variants is heterozygous in PRO1190 cells, and changes a residue (T577M) that is evolving under positive selection in primates [12]. Although additional studies will be needed to determine whether one or more of these PRO1190 PKR polymorphisms is responsible for increased resistance to RhTRS1, the results shown in Fig. 1 demonstrate that the block to VVΔEΔK+RhTRS1 replication in PRO1190 cells is mediated by PKR.

### *Rhtrs1* amplification expands virus tropism to human and rhesus macaque cells

**Figure 2:**
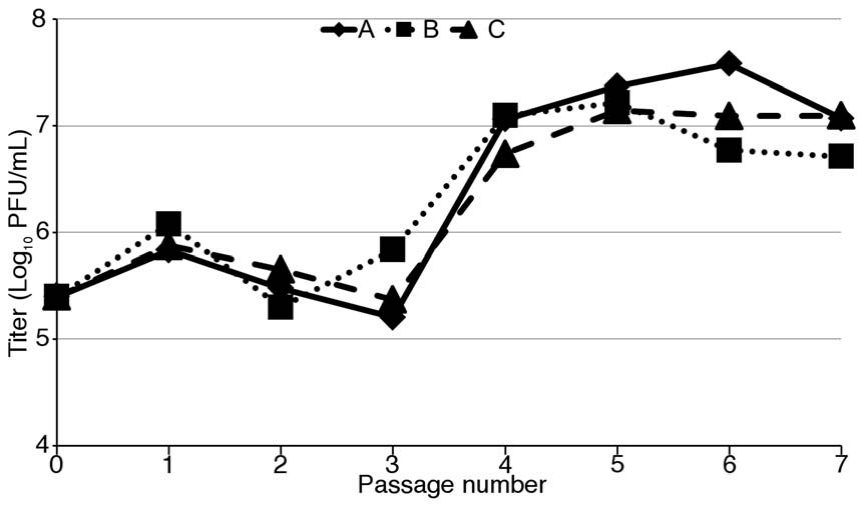
Experimental evolution of VVΔEΔK+RhTRS1 increases replication fitness in PRO1190 cells. PRO1190 cells were initially infected with VVΔEΔK+RhTRS1 (MOI = 0.1). Virus was harvested at 48 hpi, titered on BSC40 cells, and used to infect fresh PRO1190 cells and the process was repeated. Three independent infections resulted in an ∼10-fold to 100-fold gain of replication fitness in PRO1190 that was evident by passage four.

To determine whether VVΔEΔK+RhTRS1 could adapt to overcome the PKR-mediated block to replication in PRO1190 cells, we utilized a system of experimental evolution. We infected PRO1190 cells with VVΔEΔK+RhTRS1 at a low multiplicity of infection (MOI = 0.1). 48 hours post infection (hpi) we lysed the infected cells, titered the resulting virus, and infected new PRO1190 cells, repeating this cycle multiple times. After four passages we observed a 10- to 100-fold increase in viral replication that remained stable for at least three subsequent passages in each of three independent lineages (Fig. 2). We next performed a competition assay to assess the relative fitness of the passaged virus in comparison to the initial VVΔEΔK+RhTRS1 virus. We co-infected PRO1190 cells with either VVΔEΔK+RhTRS1 or VV-A, both of which express eGFP, and the same competitor, VVΔE3L+RhTRS1, which expresses β-gal (MOI = 0.1 for each virus). Virus produced 48 hpi was titered on permissive BSC40 cells and the specific progeny viruses were enumerated by detecting β-gal (VVΔE3L+RhTRS1) and eGFP (VVΔEΔK+RhTRS1 and VV-A) expression in the plaques (Fig. S3). VVΔEΔK+RhTRS1 replicated ∼3-fold better than VVΔE3L+RhTRS1, whereas VV-A replicated 290-fold better than VVΔE3L+RhTRS1, confirming that serial passage through PRO1190 cells increased the fitness of passaged viruses approximately 100-fold relative to the initial VVΔEΔK+RhTRS1.

**Figure 3:**
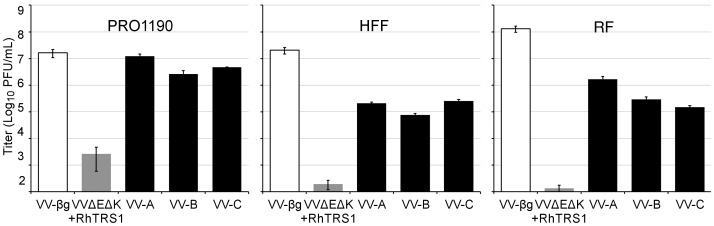
Adaptation in minimally-permissive PRO1190 cells expands the species tropism of VVΔEΔK+RhTRS1. PRO1190 (left), HFF (center), or RF (right) were infected with the indicated viruses (MOI = 0.1). Lysates were harvested 48 hpi and titered on BSC40 cells. Data are represented as means +1 STD.

Since the passaged viruses replicated more efficiently in a minimally permissive cell line, we investigated the ability of the passaged viruses to replicate in primary cells from more divergent primates. We have shown that RhTRS1 does not inhibit human or rhesus PKR in the context of VVΔE3L [20]. To determine whether the adaptations that evolved during serial passage in PRO1190 cells affected the virus species tropism, we infected primary human foreskin fibroblasts (HFF) or primary rhesus fibroblasts (RF) with VVΔEΔK+RhTRS1, each of the three passaged virus pools or VV-βg at an MOI of 0.1 (Fig. 3). As expected, VVΔEΔK+RhTRS1 replicated poorly and VV-βg replicated efficiently in each cell type. Remarkably, all three passaged pools replicated between 1000- to 10,000-fold better than VVΔEΔK+RhTRS1 in both HFF (Fig. 3, center) and RF (Fig. 3, right). For each pfu of VV-A, VV-B and VV-C used to infect the cells, 5.2, 1.9, and 6.3 pfu of progeny emerged from HFF and 40.8, 7.3, and 3.7 emerged from RF, respectively, suggesting that these viruses were sufficiently well adapted to enable continuous propagation in these cells (see below). However, these virus pools still replicated 10- to 100-fold better in PRO1190 than in either human or rhesus cells (Fig. 3, left). Thus, adaptation of VVΔEΔK+RhTRS1 in minimally permissive AGM fibroblasts also provides a substantial replication benefit in human and rhesus cells expressing distantly related PKR proteins. To elucidate the underlying mechanism for this gain of fitness, we harvested DNA from passage 7 viruses for genetic analyses and then passaged the viruses once more in PRO1190 to generate viral stocks for biochemical and infectivity analyses.

**Figure 4:**
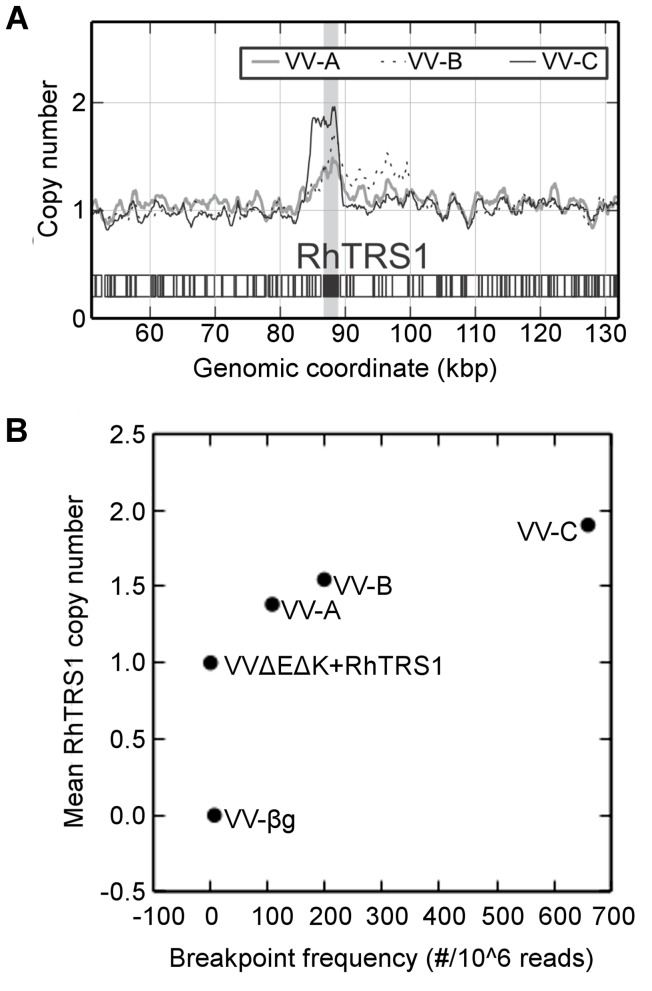
*Rhtrs1* copy number variation relative to VVΔEΔK+RhTRS1. (A) Relative read depth of Illumina sequencing reads in the passaged virus pools normalized to VVΔEΔK+RhTRS1. The graph is centered on the *rhtrs1* locus (grey bar). (B) Copy number of RhTRS1 (rescaled so that parental strain = 1) plotted against breakpoint read frequency (per million mapped reads) demonstrates agreement between each independent measure of RhTRS1 duplication.

Gene amplification as a mechanism of rapid adaptation in vaccinia virus has been well documented [17], [18], [21]. To determine whether gene amplification could account for the broadly improved replication of passaged VVΔEΔK+RhTRS1 we performed paired-end Illumina based deep sequencing (Short Read Archive #SRP033208). Based on read depth, we detected duplication of the *rhtrs1* locus in all three passage 7 virus pools but not in VVΔEΔK+RhTRS1 (Fig. 4A). Each of the passaged pools contained between 1.4 and 1.9 copies of *rhtrs1* per genome, although these numbers reflect averages of a heterogeneous population of viral genomes. Confirming this estimate of *rhtrs1* copy number, the frequency of reads in which we detected a recombination site near the *rhtrs1* locus increased as a percentage of total reads in viruses predicted to have more copies of *rhtrs1* (Fig. 4B).

We used PCR to confirm that the *rhtrs1* locus was amplified, using externally directed primers specific to *rhtrs1* that only amplify a product if there is a tandem duplication of the gene (Fig. S4A). We detected 3 kb products in all three virus pools, and 2.3 kb and 1.8 kb products only in the VV-A virus pool (Fig. S4B). We were unable to obtain enough of the 2.3 kb band for further analysis, but we did characterize the 3 kb and 1.8 kb products by Sanger sequencing. The larger product was identical in all three passaged virus pools, and represents a recombination between the vaccinia virus gene L5R upstream of *rhtrs1* with J2R downstream of *rhtrs1*. In the smaller product J2R recombined with the *neoR* gene, which was introduced as a selection marker during construction of VVΔEΔK+RhTRS1 (Fig. S3C). These two sites represented the predominant recombination sites (85.5% and 2.8% respectively) identified by Illumina deep sequencing. However, we found additional minor recombination sites by Illumina deep sequencing, including a 15 kb duplication in VV-B. The presence of an identical recombination site in all three passaged virus pools suggests that duplication may have been present at a very low frequency in the initial virus population even though we did not detect it in the Illumina sequencing data. Regardless, taken together these data demonstrate that the copy number of *rhtrs1* in the viral genome was substantially increased by passage through PRO1190 cells.

Unlike a previous study which identified adaptive point mutations arising after locus expansion [17], we did not detect any point or indel mutations in *rhtrs1* in any of the passaged virus pools. However, we identified 13 vaccinia virus gene mutations present at greater than 5% frequency in at least one of the pools (Table 1). All three passaged pools had one or more of four different single nucleotide deletions within the A35R gene at frequencies ranging from 12 to 42%. Transition mutations affecting the A24R and A37R genes were present at >50% frequencies in VV-A and VV-B respectively, but were rare or absent in the other viral pools. None of the other mutations were detected in all three pools or occurred at >50% frequency in any pool, so are unlikely to account for the improved replication of the passaged viruses. However, the presence of these VV gene mutations raised the question of whether the expanded species tropism we observed was due to the VV gene mutations or to *rhtrs1* amplification.

**Table 1:** Mutations identified in VVΔEΔK+RhTRS1 and passaged viruses relative to vaccinia virus (strain Copenhagen).

					VVΔEΔK+RhTRS1		VV-A		VV-B		VV-C	
Position	Reference Base(s)	Variant Base(s)	Gene	Effect	Read Depth	Allele Frequency	Read Depth	Allele Frequency	Read Depth	Allele Frequency	Read Depth	Allele Frequency
39202	T	TC	F7L	indel	41	4.9%	43	7.0%	47	10.6%	65	6.2%
39203	A	C	F7L	mis:N33K:aat>aaG	40	7.5%	46	19.6%	47	17.0%	65	7.7%
86598	C	T	NA	N/A*	59	1.7%	111	10.8%	124	1.6%	190	3.7%
92103	G	T	J6R	mis:G268C:ggc>Tgc	54	0.0%	118	0.0%	132	31.1%	136	0.0%
142469	C	T	A24R	mis:T1121M:acg>aTg	64	0.0%	100	73.0%	104	0.0%	133	0.0%
146037	T	TAA	A31R	indel	70	0.0%	96	17.7%	94	0.0%	134	0.0%
148449	TA	T	A35R	indel	74	0.0%	125	40.0%	132	0.0%	175	0.0%
148499	CA	C	A35R	indel	57	0.0%	117	41.9%	124	0.0%	149	1.3%
148601	CTA	C	A35R	indel	72	0.0%	93	0.0%	91	0.0%	152	7.9%
148625	TG	T	A35R	indel	77	0.0%	90	1.1%	107	12.2%	165	37.0%
149912	G	A	A37R	mis:S116N:agc>aAc	71	0.0%	125	0.0%	106	50.9%	141	2.1%
150258	C	CA	A ORF O	indel	73	0.0%	138	0.7%	125	2.4%	151	20.5%
178360	TA	T	NA	N/A*	45	0.0%	79	5.1%	75	0.0%	125	2.4%

* Not annotated.

**Figure 5:**
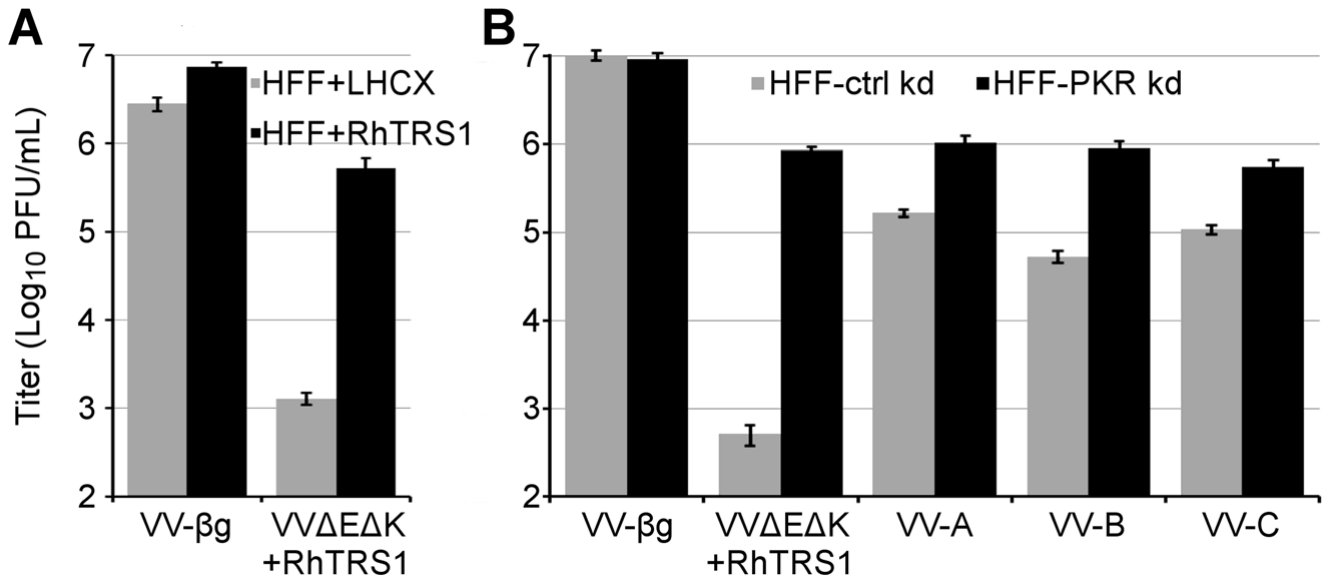
RhTRS1 overexpression or PKR knockdown permits efficient VVΔEΔK+RhTRS1 replication in HFF. (A) Viral production 48 hpi of control (HFF+LHCX, grey bars) or HFF expressing RhTRS1 (HFF+RhTRS1, black bars) with the indicated viruses (MOI = 0.1), determined by titering on HFF+TRS1. (B) Viral production 48 hpi of control (HFF-ctrl kd) or HFF with PKR knocked down by shRNA (HFF-PKR kd) with the indicated viruses (MOI = 0.1), determined by titering on BSC40 cells. Data are represented as mean +1 STD.

If *rhtrs1* amplification alone is sufficient for the observed increase in fitness, we reasoned that overexpression of *rhtrs1* in *trans* might rescue VVΔEΔK+RhTRS1 replication. To investigate this possibility, we stably transduced *rhtrs1* into HFF (HFF+RhTRS1), and confirmed RhTRS1 expression by immunoblot (data not shown). We also prepared a control cell line (HFF-LHCX) by transducing the empty vector, LHCX, into HFF. In the control cells, VVΔEΔK+RhTRS1 replicated approximately 1000-fold less efficiently than VV-βg (Fig. 5A). In HFF+RhTRS1, VVΔEΔK+RhTRS1 replication increased more than 100-fold. Thus, combined expression of RhTRS1 from genes in both the cell and the infecting virus potentiated VVΔEΔK+RhTRS1 replication, supporting the hypothesis that *rhtrs1* amplification alone is sufficient to expand the species tropism of VVΔEΔK+RhTRS1.

Although *rhtrs1* amplification provided a substantial growth benefit in HFF and RF, the passaged viruses still replicated 100- to 1000-fold less efficiently than VV-βg (Fig. 3). To determine whether this incomplete rescue in HFF was due to incomplete PKR inhibition or represented a second block to replication, we infected HFF stably transduced with either a PKR specific shRNA (HFF-PKR kd) that reduces PKR expression >95%, or a non-specific shRNA (HFF-ctrl kd) [22]. Knocking down PKR increased VVΔEΔK+RhTRS1 replication ∼1000-fold, indicating that PKR is a major barrier to replication in these cells (Fig. 5B). All three passaged virus pools replicated ∼10-fold better in HFF-PKR kd cells than in HFF-ctrl kd cells, suggesting that *rhtrs1* amplification, which fully inhibits PRO1190 PKR, only partially inhibits human PKR. However, these viruses all replicated ∼10-fold less well than VV-βg in the HFF-PKR kd cells. This remaining replication defect may be due to incomplete PKR knockdown in these cells [20], although it is also possible that an additional host factor inhibits VVΔEΔK+RhTRS1 replication in HFF.

### RhTRS1 inhibits the PKR pathway after autophosphorylation in multiple species

**Figure 6:**
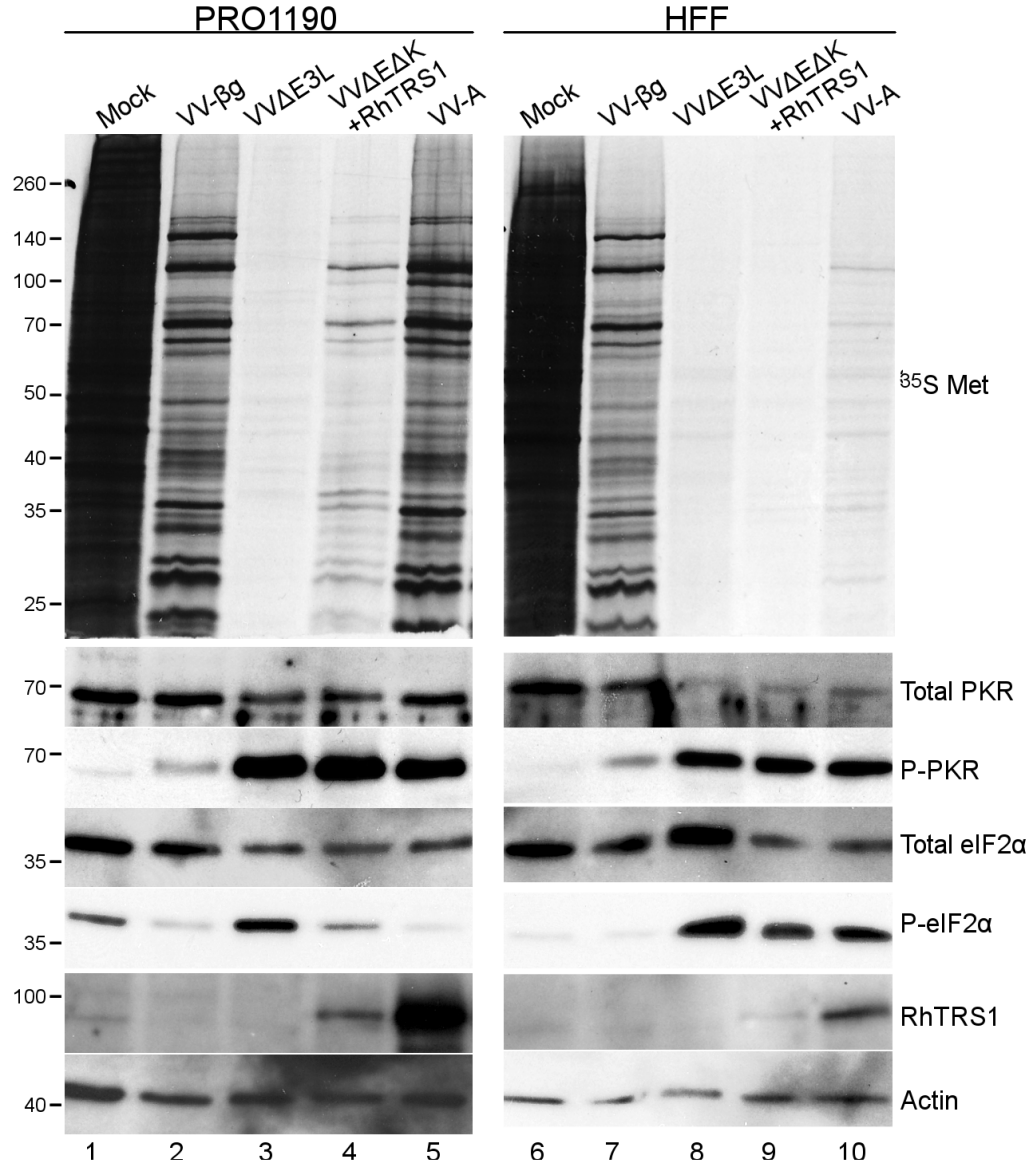
Effects of RhTRS1 overexpression on the PKR pathway in PRO1190 or HFF. PRO1190 (left) or HFF (right) were infected with the indicated viruses (MOI = 3) for 24 hpi. Proteins were labeled with ^35^S-methionine for 1 h and cell lysates were analyzed by autoradiography (top panel) or immunoblotting with the indicated antibodies (bottom panels).

Unlike many known PKR inhibitors that block PKR phosphorylation [10], [23], RhTRS1 inhibits the PKR pathway after PKR phosphorylation but prior to eIF2α phosphorylation [20]. However, it is possible that RhTRS1 amplification inhibits PKR through an alternative mechanism, such as dsRNA sequestration. To determine whether *rhtrs1* amplification altered the mechanism of PKR inhibition, we infected PRO1190 with VV-βg, VVΔE3L, VVΔEΔK+RhTRS1, and VV-A at an MOI of 3, and collected cell lysates 24 hpi. ^35^S metabolic labeling demonstrated that VVΔEΔK+RhTRS1 expressed vaccinia virus proteins in PRO1190 cells, though in much lower quantities than VV-βg, consistent with the former virus being unable to inhibit PKR completely (Fig. 6, top left panel). In contrast, VV-A produced abundant vaccinia virus proteins, similar to VV-βg, confirming that this virus efficiently inhibits the PKR pathway.

We used immunoblot analyses to determine the stage of the PKR pathway inhibited by each virus (Fig. 6, lower panels). RhTRS1 expression was noticeably higher in the VV-A infected cells compared to those infected with VVΔEΔK+RhTRS1, consistent with the better replication of VV-A in PRO1190 cells (Fig. 6, lanes 4 and 5). PKR phosphorylation was elevated in all virus infected cells except VV-βg, consistent with our previous report [20] that even when RhTRS1 blocks the PKR pathway, it does not block PKR autophosphorylation (Fig. 6, lanes 3–5). Phospho-eIF2α levels in VVΔEΔK+RhTRS1 infected cells were intermediate between mock and VVΔE3L infected PRO1190 cells, suggesting that a single RhTRS1 gene weakly inhibits the PKR pathway (Fig. 6, lane 4). Infection with VV-A resulted in low levels of eIF2α phosphorylation, similar to that detected in VV-βg infected PRO1190 cells (Fig. 6, compare lanes 2 and 5), indicating that RhTRS1 amplification is sufficient to completely inhibit PKR-mediated translational shutdown at a stage after PKR phosphorylation. Together, these data suggest that PKR-mediated pressure in PRO1190 cells selected for rapid amplification of the *rhtrs1* locus, and that this amplification was sufficient to enable the virus to block PKR-mediated defenses in PRO1190 cells without altering the mechanism of RhTRS1-mediated PKR inhibition.

In previous studies, RhTRS1 alone was insufficient to inhibit human PKR [20]; however, the passaged viruses reported here replicate substantially better in HFF than VVΔEΔK+RhTRS1, suggesting that amplification of *rhtrs1* is able to inhibit PKR at least partially in these cells. To test this hypothesis and determine whether the mechanism of PKR inhibition was the same in HFF as it is in PRO1190, we infected HFF (MOI = 3) and prepared cell lysates 24 hpi. In contrast to infection of PRO1190 cells, infection of HFF with VVΔEΔK+RhTRS1 resulted in nearly complete shut off of protein synthesis by 24 hpi (Fig. 6, top right panel) and produced only trace amounts of RhTRS1. Compared to VVΔEΔK+RhTRS1, VV-A infection of HFF resulted in detectable, though still low overall levels of ^35^S labeled proteins, and much more RhTRS1 (Fig. 6, lanes 9 and 10). PKR phosphorylation and eIF2α phosphorylation were elevated after infection with both VVΔEΔK+RhTRS1 and VV-A compared to mock or VV-βg controls (Fig. 6, compare lane 9 to lanes 6 and 7). These data suggest that a single copy of *rhtrs1* was insufficient to inhibit the translational shutoff mediated by human PKR, but amplification of this weak antagonist resulted in partial inhibition of human PKR allowing enough protein synthesis to support a modest level of virus replication.

### RhTRS1 amplification in PRO1190 is a necessary intermediate for VVΔEΔK+RhTRS1 replication in HFF

**Figure 7:**
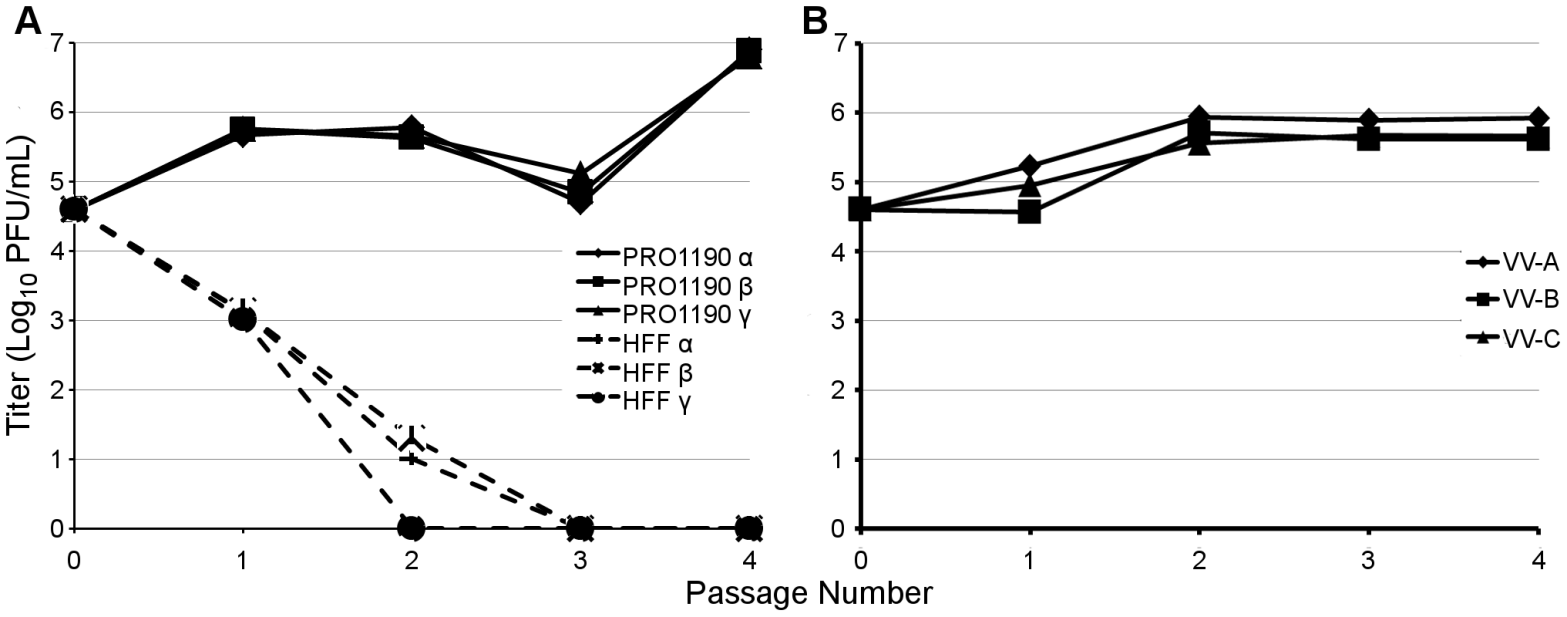
PRO1190-adapted viruses, but not VVΔEΔK+RhTRS1, stably replicate in HFF. (**A**) PRO1190 or HFF were infected with VVΔEΔK+RhTRS1 (MOI = 0.1), for 48 h, after which viral progeny were collected, titered on BSC40 cells, and used to infect new cells (MOI = 0.1, or the full volume of the harvested lysate when too little virus was obtained). Three independent infections resulted in a >10-fold gain of replication fitness in PRO1190 over the course of the experiment, but no virus survived three rounds of passage through HFF. (B) HFF were infected with PRO1190-adapted VV-A, VV-B, or VV-C virus pools (MOI = 0.1). Virus progeny harvested at 48 hpi was titered on BSC40 cells and used to infect new HFF and the process was repeated. All three virus pools replicated in HFF for at least four rounds of serial passage, and acquired an additional, modest gain in replication fitness.

Because selection in AGM cells resulted in a broad expansion of viral species tropism, we investigated whether passage of VVΔEΔK+RhTRS1 directly in HFF would similarly select for mutants, such as *rhtrs1* amplification, that improved replication in HFF. We therefore serially infected PRO1190 cells and HFF with VVΔEΔK+RhTRS1 in parallel (Fig. 7). In PRO1190 cells, viral fitness again increased after four passages. In contrast, virus replication was strongly inhibited in HFF, and we were unable to detect any viral replication after three rounds of infection in all three pools. These data suggest that, under these experimental conditions, adaptation in PRO1190 cells was a necessary intermediate step for improved replication in HFF.

Finally, we evaluated whether the improved replication of the viruses that had been passaged in PRO1190 cells was sufficient to enable stable propagation in HFF. We therefore serially infected HFF with VV-A, VV-B and VV-C at low multiplicity of infection (MOI = 0.1 at each passage). We were able to propagate these viruses in HFF for at least four passages. Moreover, replication increased between 5- to 14-fold after only two passages, suggesting further adaptation occurred in HFF cells (Fig. 7B). To define mutations that may have evolved during serial passage in HFF, we performed paired-end Illumina based deep sequencing on viral DNA isolated after the fourth round of passage in HFF. Again, we did not find any mutations in *rhtrs1*; however, we detected an average *rhtrs1* copy number of 1.7, 2.8, and 2.7 for the virus pools derived from VV-A, VV-B and VV-C, respectively, representing an average expansion of the *rhtrs1* locus by approximately one additional copy relative to the PRO1190-adapted viruses. In addition, two VV gene mutations (in F7L [indel] and J6R) that arose during PRO1190 adaptation were lost after HFF adaptation, and two new mutations (in F7L [missense] and H4L) of uncertain significance appeared during HFF adaptation (Table S3). Taken together, our study suggests the hypothesis that gene amplification acts broadly to increase replication in a variety of hosts, and may provide a molecular foothold that allows for continued species-specific adaptation of the virus in more resistant hosts.

## Discussion

Cross-species pathogen transmissions have been responsible for more than 60% of all emerging infectious diseases in humans during the past 70 years [24]. Human immunodeficiency viruses, avian influenza viruses, and the recently described Middle East respiratory syndrome coronavirus exemplify the ongoing threat and potential of animal viruses to spread to and among humans, highlighting the urgent need to understand the mechanisms underlying cross-species transmission and adaptation to new hosts. One such mechanism, genetic locus amplification in response to selective pressure, has been observed in both viruses and bacteria [16]–[18], [21]. Here we demonstrate that amplification of the exogenous gene *rhtrs1* is sufficient to block potent PKR-mediated inhibition and improve VVΔEΔK+RhTRS1 replication in AGM-derived PRO1190 cells. This adaptation also expanded the species tropism of the virus, enabling markedly improved replication in otherwise resistant human and rhesus monkey cells. Importantly, VVΔEΔK+RhTRS1 failed to replicate in HFF to a level sufficient to sustain transmission upon serial passage, demonstrating that adaptation in PRO1190 was a critical intermediate step to expand the viral species tropism. Thus, the process of adaptation in one host may increase the likelihood of virus transmission to a variety of divergent species.

Under PKR-mediated selective pressure, the *rhtrs1* locus amplified during serial passage of VVΔEΔK+RhTRS1 in minimally permissive PRO1190 cells (Fig. 4). It is not clear whether the initial duplication(s) occurred during preparation of the VVΔEΔK+RhTRS1 stock in BSC40 cells or during the first few passages in the PRO1190 cells. The observation of a faint PCR product from the starting virus, VVΔEΔK+RhTRS1 using outward directed *rhtrs1* primers in one of three experiments (Fig. S4), and the detection of an identical recombination break point in all 3 independently passaged virus pools suggest the amplification may have been present at a very low level in the starting virus. However, amplification of the locus in VVΔEΔK+RhTRS1 occurred below the level of Illumina deep sequencing detection (Fig. 4), supporting the idea that if *rhtrs1* duplications are present they are rare in the initial VVΔEΔK+RhTRS1 stock. Additionally, we detected recombination between J2R and *neoR* (Fig. S4) only in the VV-A pool, suggesting that recombination events did arise during serial passage. Regardless of when they arose, amplifications of the *rhtrs1* locus were substantially enriched during virus passage under selective pressure (Figs. 2 and 4). A previous study demonstrated amplification of K3L as an adaptive mechanism against human PKR with strikingly similar kinetics to the current study [17]. Together, these two studies support the hypothesis that preexisting or frequently arising *de novo* gene duplications enable vaccinia virus to adapt rapidly to selective conditions imposed by relatively resistant host restriction factors.

The “accordion hypothesis” of rapid evolution posits that gene amplification provides a replication benefit to the virus and those extra copies of a weak viral antagonist of host defenses provide additional templates to acquire potentially adaptive mutations. Indeed, Elde, *et al.* detected such an adaptive mutation in K3L (H47R), apparently arising after amplification of the locus [17]. Therefore, we were surprised that no mutations arose in *rhtrs1*, although it may be that additional rounds of replication would reveal such mutations. We did, however, detect 13 mutations in endogenous vaccinia virus genes that occurred at >5% frequency (Fig. 4). While our data suggest that none of these mutations are necessary to expand the species tropism of VVΔEΔK+RhTRS1 (Fig. 5), we have not ruled out the possibility that they may provide some replication benefit. The presence of mutations in A24R, A35R, and A37R are the most intriguing, as they were either present at >50% frequency in one pool (A24R and A37R) or detectable in all three pools (A35R). None of these genes has been previously implicated as a PKR antagonist. A37R is conserved across multiple poxvirus families (http://www.poxvirus.org), but its function is unknown. A24R is a subunit of RNA polymerase. If the mutation we identified acts like some other reported RNA polymerase mutations to decrease transcription elongation [25], [26], this mutation might result in less dsRNA production and therefore less PKR activation. A35R is a gene of unknown biochemical function that may be involved in evasion of the adaptive immune response [27], [28]. All three passaged virus pools contained nucleotide deletions in A35R at greater than 10% frequency. A35R orthologs are conserved across most poxviruses but, intriguingly, variola virus contains a truncation in its A35R gene, demonstrating that similar truncations have evolved in the past. Further studies are underway to evaluate the potential contributions of these mutations to viral replication.

Consistent with previous studies of RhTRS1 in other AGM cell types [20], PKR, but not eIF2α, was phosphorylated in PRO1190 cells infected with VV-A. Thus, the improved replication of the passaged virus pools in PRO1190 cells was likely due to enhancement of the basic RhTRS1-mediated inhibition of PKR, and not due to another mechanism, such as dsRNA sequestration or reduction in the abundance of dsRNA. VVΔEΔK+RhTRS1 infected PRO1190 cells had phospho-eIF2α levels lower than that found in VVΔE3L infected cells (Fig. 6, lanes 3 and 4), suggesting that even just a single copy of *rhtrs1* is able to inhibit PKR function to a small degree, but amplification of the locus appears to be needed to express enough RhTRS1 to inhibit eIF2α phosphorylation potently and enable efficient viral replication. It is also possible that elevated expression of RhTRS1 is necessary to block another activity of PKR, such as autophagy or inflammasome responses [29]–[31] that might aid in replication.

Adaptation of VVΔEΔK+RhTRS1 to PRO1190 cells provided a substantial replication advantage in both human and rhesus monkey fibroblasts. Although VV-A replicated to much higher titers and expressed more RhTRS1 than VVΔEΔK+RhTRS1 in HFF, ^35^S metabolic labeling revealed relatively low protein synthesis rates and eIF2α phosphorylation was still elevated after VV-A infection compared to VV-βg. A substantial proportion of the block to VV-A replication in HFF is still mediated by PKR despite RhTRS1 overexpression (Fig. 5B), suggesting that further adaptation in HFF may be necessary to block the PKR pathway in HFF completely. These results indicate that RhTRS1 overexpression blocks human PKR incompletely. Nonetheless, viruses that had adapted by initial passage in PRO1190s replicated in HFF at a level sufficient to enable sustained passage in HFF (Fig.7B). Furthermore, these PRO1190-adapted viruses acquired additional changes as a result of serial passage in HFF, although the biological relevance of these changes is currently unclear. Combined with the observation that serial passage of VVΔEΔK+RhTRS1 in HFF failed to generate any adapted viruses (Fig. 7A), these data suggest that adaptation of *rhtrs1* in minimally permissive PRO1190 cells was a critical intermediate step in the generation of a virus with broadened species tropism.

Cross-species pathogen transmission is an important source of emerging infections worldwide. Our study illustrates that gene amplification of a weak viral antagonist of PKR can broaden the host range of vaccinia virus. The presence of gene families in other large DNA viruses, e.g. the cytomegalovirus US22 family, of which *rhtrs1* is a member, provides indirect evidence that episodes of locus amplification have also occurred in other viruses. Adaptation of a viral antagonist through non-synonymous mutations has the potential to confer a species-specific advantage for the virus in a specific host. In contrast, gene amplification and subsequent over-expression of the antagonist is more likely to increase protein activity through mass action effects in a variety of hosts. Thus, gene amplification may be a common evolutionary strategy employed by large DNA viruses, permitting modest replication in otherwise resistant host species and providing a molecular foothold that enables further adaptations necessary for more efficient replication and spread in the new host.

## Materials and Methods

### Viruses and cell culture

AGM fibroblasts (PRO1190, Coriell Institute for Medical Research) human foreskin fibroblasts (HFF), rhesus fibroblasts (RF), BSC40 and HeLa cells, and derivative cell lines were maintained in Dulbecco's modified Eagle's medium supplemented with 10% NuSerum (BD Biosciences) as previously described [32]. At times, PRO1190 cell lines were also propagated in Minimal Essential Medium with 20% fetal calf serum and anitbiotics to enable more rapid growth. PRO1190 cells were transduced with a nonsilencing control or PKR-targeting shRNA lentiviral vectors (Open Biosystems, catalogue numbers RHS4430-98819555 and RHS4346, respectively) and selected in puromycin (5 µg/ml) to generate PRO1190-ctrl k/d and PRO1190-PKR k/d cells. pEQ1364 was constructed by moving the RhTRS1 gene, with a C-terminal biotinylation signal and 6x-His tag, as a HindIII/PmeI fragment from pEQ1215 [20] into the HindIII/HpaI sites of pLHCX (Clontech Laboratories, Inc). HFF-LHCX and HFF+RhTRS1 were produced by transducing HFF with retroviral vectors made using LHCX and pEQ1364, respectively and selecting with hygromycin B (100 µg/ml).

Vaccinia virus (VV) Copenhagen strain (VC2) [33] and VVΔE3L [34], both obtained from Bertram Jacobs (Arizona State University), and VV-βg (VC2-LacZ in [20] were propagated and titered in BSC40 cells.

VC-R2 (VVΔE3LΔK3L) was constructed by replacing the E3L gene in the K3L-deleted VACV vP872 strain (ΔK3L in VC2 background, provided by Bertram Jacobs) [35] by homologous recombination. The 518 bp 5′ arm was created by PCR amplification of VC2 DNA with primers C15 (5′-GATTAAGGGTACTAGCGGCACCG′3′)×C16 (5′-TTTTAGAGAGAACTAACACAACCAGC-3′). The 512 bp 3′ arm was created by PCR amplification of VC2 DNA with primers C19 (5′-GTGTAGTAAGCTAGCGAGCTCGGTACCTTCTAGTTATCAATAACAGTTAGTAGTTTAG-3′)×C20 (5′-CCAACAAACTGTTCTCTTATGAATCG-3′). The reading frame of EGFP including the PEST sequence was amplified with primers C17 (5′-GCTGGTTGTGTTAGTTCTCTCTAAAACCCGGGATCCACCGGTCGCC-3′)×C18 (5′-GGTACCGAGCTCGCTAGCTTACTACACATTGATCCTAGCAGAAGC-3′) using pD2EGFP-N1 (Clonetech) as the template. PCR products were gel-purified and mixed together as template for fusion PCR using C15×C20. PfuUltra polymerase (Agilent Technologies) was used for these PCR reactions. PCR products were cloned into the pCR2.1 TOPO vector to generate plasmid S96. S96 was used as template for PCR amplification of marker +5′ and 3′ arms using C15×C20 followed by gel-purification. BS-C-1 cells grown on 12 well plates were infected with vP872 at MOI = 2 and transfected 2 hours after infection with 1 µg of the purified PCR product. Cell lysates were collected after 18 hours and plated in a dilutions series on RK13+E3L+K3L cells [36]. Green plaques were picked after 48 hours at the highest dilution possible and plaque purified an additional three times on RK13+E3L+K3L cells. EGFP expression in VC-R2 is under the control of the endogenous E3L promoter. VVΔEΔK was propagated and titered using HFF+TRS1 cells (HF-TRS1 in [37]).

VVΔEΔK+RhTRS1 was constructed by homologous recombination of plasmid pEQ1233 [20] into the thymidine kinase (TK) locus of VVΔEΔK. Recombinant virus was plaque purified three times in BSC40 under G418 selection and subsequently propagated and titered on BSC40 cells.

### PRO1190 PKR sequence analysis

Total RNA from PRO1190 cells was amplified using previously reported PKR specific primers [12]. The amplification product was gel purified and cloned using the StrataClone PCR cloning kit (Agilent). Multiple plasmids were submitted for Sanger sequencing using PKR specific sequencing primers (#859: 5′-ATGGCTGGTGATCTTGCAC; #860: 5′-GTGAACAACTCACTTGCTTC; #861: 5′-GAAACTAGACAAAGTTTTGGC; #862: 5′-CTAACATGTATGTCGTTCCT; #863: 5′-AAGGCACTTAGTCTTTGATC; #864: 5′-TCTGATATCTCAAGCAATGC). Contigs were assembled and curated in Geneious Pro v4.8.5 (GenBank #KF728076-7). The predicted amino acid sequence was aligned to the predicted amino acid sequences of previously reported AGM (GenBank # EU733254), rhesus macaque (GenBank# EU733261), and human PKR (GenBank # NM001135651) using ClustalW2 (http://www.ch.embnet.org/software/ClustalW.html) [38].

### Experimental evolution of VVΔEΔK+RhTRS1

For every round of infection, triplicate confluent 10 cm dishes (Figure 2) or 6-well plates (Figure 7) of PRO1190 or HFF were infected with VVΔEΔK+RhTRS1 (MOI = 0.1). Two days post-infection, cells were collected, pelleted and resuspended in 1 mL DMEM+10% NuSerum. After three freeze/thaw cycles, virus titers were determined on BSC40 by plaque assays and used for the next round of infection. If insufficient virus was produced to infect cells at MOI = 0.1, the entire volume of virus lysate was used in the subsequent round of infection.

Vaccinia virus DNA from passage 7 in PRO1190 (Figure 2) or passage 4 in HFF (Figure 7 right) viruses was purified from infected cell cytoplasmic extracts for genetic analyses described below [39]. The passage 7 pools were further expanded (passage 8) in PRO1190 for use in virologic assays.

### Competition assay

PRO1190 cells were co-infected with 0.1 MOI of either VVΔEΔK+RhTRS1 or VV-A, and 0.1 MOI of VVΔE3L+RhTRS1 as a common competitor. Two days post-infection cells were collected, pelleted and resuspended in 1 mL DMEM+10% NuSerum. After three freeze/thaw cycles, virus titers were determined on BSC40 by plaque assays. VVΔE3L+RhTRS1 plaques were detected with the β-gal substrate ImaGene Red C_12_RG following the manufacturer's directions (Life Technologies). Plaques were imaged on a Typhoon Trio imager (eGFP - 488 nm excitation, 520 BP 40 filter; ImaGene Red - 532 nm excitation, 580 BP 30 filter) at 50 µm/pixel resolution, and classified as GFP+, ImaGene Red+, or double positive using ImageJ software (http://rsb.info.nih.gov/ij/).

### Genomic analyses

#### Externally directed PCR

Externally directed oligonucleotides were designed that bind near the 5′ or 3′ end of RhTRS1 (#915: 5′-TGTGGGAGGATGCATTGCAG; #916: 5′-GGCGACTACAATCCCCATTG). High-fidelity PCR was performed with 100 ng of purified virus DNA following the manufacturer's directions (Phusion, Thermo Scientific). Reactions were pulsed at 98°C×30 s followed by 25 cycles of 98°C×10 s, 65°C×20 s, 72°C×3 min. Amplification products were gel-purified and cloned using the Strataclone PCR cloning kit (Agilent) following the manufacturer's suggested protocol. Recombination sites were identified by Sanger sequencing of these products.

#### Library preparation and sequencing

From each viral pool, 75 ng of viral DNA was sheared and ligated to adaptors by transposition using the Nextera kit (Epicentre), following the manufacturer's direction with several modifications: transposition was performed in 20 ul volume using 0.1 µl Nextera transposase enzyme and “HMW” reaction buffer at final 1X. Transposition was carried out for 10 minutes at 55°C, after which sheared, adaptor-ligated templates were transferred directly to PCR reactions for amplification. In addition, PCR incorporated a sample-specific barcode tag on the reverse primer. Amplified libraries were pooled and cleaned using Ampure beads (Beckman Coulter) at a 1.8∶1 ratio of beads to input. The pooled libraries were sequenced on an Illumina HiSeq 2000 instrument (Illumina) using 101-bp forward and reverse reads with a 9-bp index to read the per-sample barcode.

#### Sequence analysis

De novo assembly guided by the Vaccinia virus Copenhagen genome (GenBank #M35027.1) was used to construct a reference assembly sequence for VVΔEΔK+RhTRS1 (Tables S1 and S2). Shotgun reads from the parental virus were assembled to contigs using ABySS 1.3.4 [40]; parental virus reads were then realigned against the resulting assembly contigs using bwa 0.6.1 [41]. Discrepancies between reads and contigs, reflecting *de novo* assembly errors, were corrected in the contigs following manual examination, and contigs were ordered and concatenated by comparison to the Copenhagen sequence.

Reads from each evolved viral pool were aligned to the parental reference genome, and single-base and short-indel variants were called using the Genome Analysis Toolkit [42]. Custom scripts were used to determine the allele frequency (fraction reads supporting variant allele out of all aligned reads at a given site) for all variants found in any sample. Copy number was estimated by counting the depth of read coverage within sliding windows of 100 bp, correcting for effects of differences in G+C composition, and dividing by the parental strain read depth. Everted reads at duplication breakpoints were identified using bwasw [43] and custom scripts, as previously described [17].

### Immunoblot analyses

Cells were mock infected or infected with vaccinia viruses (MOI = 3). One day postinfection, the cells were lysed in 2% sodium dodecyl sulfate (SDS). Equivalent amounts of the lysates were separated on 10% SDS-polyacrylamide gels, transferred to polyvinylidene difluoride (PVDF) membranes, and probed with one of the following antibodies: PKR (sc-6282; Santa Cruz Biotechnology, Inc.), phospho-PKR (T446; 1120-1; Epitomics), eIF2α or phospho-eIF2α (Ser51) antibody (both from Cell Signaling Technology, catalog numbers 9722 and 9721, respectively), TRS1 α999 [37], or actin (A2066; Sigma). All purchased antibodies were used according to the manufacturer's recommendations. Proteins were detected using the Western Star chemiluminescent detection system (Applied Biosystems) according to the manufacturer's recommendations. Densitometry measurements were performed using ImageJ.

### RT-qPCR

Total RNA was extracted from PRO1190, PRO1190-ctrl kd, and PRO1190-PKR kd cells with TRIzol reagent following the manufacturer's protocol (Invitrogen), and 1 ng of total RNA was assayed per reaction. The standard curve was generated from 10-fold serial dilutions of a PRO1190 PKR containing plasmid (described above in *PRO1190 PKR sequence analysis*, pEQ1334) diluted in 200 pg/µL salmon sperm DNA. All samples were amplified in triplicate using the GoTaq 1-step RT-qPCR kit following the manufacturer's protocol (Promega) using PKR specific primers (#1063: 5′-CACAGAATTGACGGAAAGAC; #1064: 5′-ATCCCAACAGCCATTGTAGT). RT-qPCR was performed on a Rotor-Gene Q thermocycler (Qiagen) with temperature holds at 37°C×15 min and 95°C×10 min followed by 40 cycles of 95°C×10 s, 60°C×30 s. Raw data was analyzed using the included Rotor-Gene Q series software using the automatic cycle threshold (Ct) setting for assigning baseline and threshold for Ct determination. Nonlinear regression analysis to determine PKR copy number in the experimental samples was performed in GraphPad Prism 6.

### Metabolic labeling

PRO1190 and HFF cells were mock infected or infected with the indicated viruses (MOI = 3). At 24 hpi, the cells were labeled for 1 h with 100 µCi/ml L-[^35^S]methionine/L-[^35^S]cysteine (EasyTag express protein labeling mix; PerkinElmer) in medium lacking methionine and cysteine. The cells were then lysed in 2% SDS. Equivalent amounts of protein from each sample were separated on 10% SDS-polyacrylamide gels, dried, and visualized by autoradiography.

## Supporting Information

Figure S1 **RT-qPCR analysis of PKR copy number in PRO1190-derived cell lines.** RT-qPCR was performed on serial 10-fold dilutions of a PKR containing plasmid from 10^6^ to 10^1^ copies of PKR to generate a standard curve (top). Data are represented as the mean +/− the 95% confidence interval. PKR copy number/ng total RNA was interpolated from this standard curve for PRO1190, PRO1190-ctrl kd, and PRO1190-PKR kd cells (bottom). Relative to PRO1190 cells, PRO1190-ctrl kd cells expressed slightly less PKR (20% decrease), while PRO1190-PKR kd cells had a 69% decrease in PKR expression. Similarly, PRO1190-PKR kd cells expressed 60% less PKR than PKR-ctrl kd cells, consistent with Fig. 1B.
(TIF)

Figure S2 **Amino acid alignment of African green monkey, human, and rhesus PKR.** Predicted PKR amino acid sequences from AGM (PRO1190T1610 and EU733254), rhesus macaque (GenBank# EU733261), and human (GenBank # NM001135651) were aligned using CLUSTAL W (1.83) [38]. We identified three non-synonymous amino acid differences (grey or green highlighted residues) between the two AGM alleles. One of these differences is at a site that is evolving under positive selection in primates (green highlighted residue) [12]. Relative to PRO1190 PKR, rhesus and human PKR are 90.8% and 83.2% identical at the amino acid level, respectively.
(TIF)

Figure S3 **Increased fitness of VV-A compared to VVΔEΔK+RhTRS1 assessed by indirect competition assay.** PRO1190 cells were co-infected with either VVΔEΔK+RhTRS1 or VV-A (MOI = 0.1) and the same competitor virus, VVΔE3L+RhTRS1 (MOI = 0.1). Two days post-infection viral progeny were collected and titered on BSC40 cells. (A) Representative Typhoon image of virus titer plates produced by infection with VVΔE+RhTRS1 in combination with either VVΔEΔK+RhTRS1 (top panels) or VV-A (bottom panels). (B) Plaques were scored for eGFP expression (VVΔEΔK+RhTRS1 or VV-A), β-gal expression (VVΔE3L+RhTRS1) or both (double +) as described in Materials and Methods. VV-A replicated ∼100-fold better than VVΔEΔK+RhTRS1 relative to VVΔE3L+RhTRS1. Data are represented as the mean +1 SD.
(TIF)

Figure S4 **Predominant recombination sites identified in** ***rhtrs1*** **locus amplification.** (A) Schematic of externally directed PCR. Externally directed oligonucleotides (grey arrows) were designed to bind to the ends of *rhtrs1* (white). Only tandem duplications will produce an amplification product. (B) Externally directed PCR revealed enrichment of *rhtrs1* amplification products in the passaged virus pools. (C) Schematic of the initial *rhtrs1* locus in VVΔEΔK+RhTRS1 (top), and the predominant recombination sites identified in all three passaged viruses (middle), or in VV-A alone (bottom). Vaccinia virus genes are colored green or blue, and exogenous sequences are colored dark red (*rhtrs1*) or brown (*neoR*). PCR products amplified by externally directed primers (black bars), the duplicated sequence (grey boxes), and the recombination sites (red arrowheads), are indicated. Recombination sites occur (middle) after nt 84267 (J2R) and before nt 83200 (L5R) relative to the reference vaccinia virus. Copenhagen strain (Genbank #M35027.1), or (bottom) after nt 84237 (J2R) and before nt 622 (*neoR* - pcDNA3.1, Invitrogen).
(TIF)

Table S1 **Genomic coordinates for regions in whole genome alignment between Vaccinia copenhagen reference (GenBank M35027.1) and parental genome (VVΔEΔK+RhTRS1 genome) sequenced here, as determined by MUMmer 3.2.1** [**44**] **.**
(DOCX)

Table S2 **Substitution and short indel variants (<10 bp) present in parental genome (VVΔEΔK+RhTRS1) as compared to Vaccinia Copenhagen genome (excluding any in the inverted terminal repeat regions).** Predicted effects upon protein coding genes as annotated in the Copenhagen reference are listed, with more than one line per variant indicating effects upon multiple overlapping gene models.
(DOCX)

Table S3 **Mutations identified in VV-A, VV-B and VV-C after four passages through HFF relative to vaccinia virus (strain Copenhagen).**
(DOCX)

Table S4 **Accession numbers.**
(DOCX)

## References

[1] Yamada A (2004) [Zoonoses]. Uirusu 54: 17–22. 15449900 10.2222/jsv.54.17

[2] Mahy BW (2000) The global threat of emerging infectious diseases. Isr Med Assoc J 2 Suppl: 23–26. 10909413

[3] Bengis RG, Leighton FA, Fischer JR, Artois M, Morner T, et al (2004) The role of wildlife in emerging and re-emerging zoonoses. Rev Sci Tech 23: 497–511. 15702716

[4] Parrish CR, Holmes EC, Morens DM, Park EC, Burke DS, et al (2008) Cross-species virus transmission and the emergence of new epidemic diseases. Microbiol Mol Biol Rev 72: 457–470. 18772285 10.1128/MMBR.00004-08 PMC2546865

[5] Chua KB (2003) Nipah virus outbreak in Malaysia. J Clin Virol 26: 265–275. 12637075 10.1016/s1386-6532(02)00268-8

[6] Daszak P, Plowright R, Epstein JH, Pulliam J, Abdul Rahman S, et al.. (2006) The emergence of Nipah and Hendra virus: pathogen dynamics across a wildlife-livestock-human continuum. In: Collinge RS, editors. Disease ecology: community structure and pathogen dynamics. Oxford, United Kingdom: Oxford University Press. pp. 186–201.

[7] Sharp PM, Hahn BH (2011) Origins of HIV and the AIDS pandemic. Cold Spring Harb Perspect Med 1 (1) a006841. 22229120 10.1101/cshperspect.a006841 PMC3234451

[8] Stremlau M, Owens CM, Perron MJ, Kiessling M, Autissier P, et al (2004) The cytoplasmic body component TRIM5alpha restricts HIV-1 infection in Old World monkeys. Nature 427: 848–853. 14985764 10.1038/nature02343

[9] Sadler AJ, Williams BR (2007) Structure and function of the protein kinase R. Curr Top Microbiol Immunol 316: 253–292. 17969452 10.1007/978-3-540-71329-6_13

[10] Mohr IJ, Pe'ery T, Mathews MB (2007) Protein synthesis and translational control during viral infection. In: Mathews MB, Sonenberg N, Hershey JWB, editors. Translational control in biology and medicine. Cold Spring Harbor, NY: Cold Spring Harbor Laboratory Press. pp. 545–599.

[11] Daugherty MD, Malik HS (2012) Rules of engagement: molecular insights from host-virus arms races. Annu Rev Genet 46: 677–700. 23145935 10.1146/annurev-genet-110711-155522

[12] Elde NC, Child SJ, Geballe AP, Malik HS (2009) Protein kinase R reveals an evolutionary model for defeating viral mimicry. Nature 457: 485–489. 19043403 10.1038/nature07529 PMC2629804

[13] Rothenburg S, Seo EJ, Gibbs JS, Dever TE, Dittmar K (2009) Rapid evolution of protein kinase PKR alters sensitivity to viral inhibitors. Nat Struct Mol Biol 16: 63–70. 19043413 10.1038/nsmb.1529 PMC3142916

[14] Brown CJ, Todd KM, Rosenzweig RF (1998) Multiple duplications of yeast hexose transport genes in response to selection in a glucose-limited environment. Mol Biol Evol 15: 931–942. 9718721 10.1093/oxfordjournals.molbev.a026009

[15] Gonzalez E, Kulkarni H, Bolivar H, Mangano A, Sanchez R, et al (2005) The influence of CCL3L1 gene-containing segmental duplications on HIV-1/AIDS susceptibility. Science 307: 1434–1440. 15637236 10.1126/science.1101160

[16] Roth JR, Andersson DI (2004) Amplification-mutagenesis–how growth under selection contributes to the origin of genetic diversity and explains the phenomenon of adaptive mutation. Res Microbiol 155: 342–351. 15207866 10.1016/j.resmic.2004.01.016

[17] Elde NC, Child SJ, Eickbush MT, Kitzman JO, Rogers KS, et al (2012) Poxviruses deploy genomic accordions to adapt rapidly against host antiviral defenses. Cell 150: 831–841. 22901812 10.1016/j.cell.2012.05.049 PMC3499626

[18] Slabaugh M, Roseman N, Davis R, Mathews C (1988) Vaccinia virus-encoded ribonucleotide reductase: sequence conservation of the gene for the small subunit and its amplification in hydroxyurea-resistant mutants. J Virol 62: 519–527. 2826813 10.1128/jvi.62.2.519-527.1988 PMC250563

[19] Kondrashov FA (2012) Gene duplication as a mechanism of genomic adaptation to a changing environment. Proc Biol Sci 279: 5048–5057. 22977152 10.1098/rspb.2012.1108 PMC3497230

[20] Child SJ, Brennan G, Braggin JE, Geballe AP (2012) Species specificity of protein kinase r antagonism by cytomegalovirus TRS1 genes. J Virol 86: 3880–3889. 22278235 10.1128/JVI.06158-11 PMC3302489

[21] Slabaugh MB, Roseman NA, Mathews CK (1989) Amplification of the ribonucleotide reductase small subunit gene: analysis of novel joints and the mechanism of gene duplication in vaccinia virus. Nucleic Acids Res 17: 7073–7088. 2674905 10.1093/nar/17.17.7073 PMC318434

[22] Child SJ, Brennan G, Braggin JE, Geballe AP (2012) Species specificity of protein kinase r antagonism by cytomegalovirus TRS1 genes. J Virol 86: 3880–3889. 22278235 10.1128/JVI.06158-11 PMC3302489

[23] Garcia MA, Meurs EF, Esteban M (2007) The dsRNA protein kinase PKR: virus and cell control. Biochimie 89: 799–811. 17451862 10.1016/j.biochi.2007.03.001

[24] Jones KE, Patel NG, Levy MA, Storeygard A, Balk D, et al (2008) Global trends in emerging infectious diseases. Nature 451: 990–993. 18288193 10.1038/nature06536 PMC5960580

[25] Bayliss CD, Condit RC (1993) Temperature-sensitive mutants in the vaccinia virus A18R gene increase double-stranded RNA synthesis as a result of aberrant viral transcription. Virology 194: 254–262. 8480421 10.1006/viro.1993.1256

[26] Cresawn SG, Prins C, Latner DR, Condit RC (2007) Mapping and phenotypic analysis of spontaneous isatin-beta-thiosemicarbazone resistant mutants of vaccinia virus. Virology 363: 319–332. 17336362 10.1016/j.virol.2007.02.005 PMC1950264

[27] Rehm KE, Connor RF, Jones GJ, Yimbu K, Roper RL (2010) Vaccinia virus A35R inhibits MHC class II antigen presentation. Virology 397: 176–186. 19954808 10.1016/j.virol.2009.11.008 PMC2813887

[28] Rehm KE, Roper RL (2011) Deletion of the A35 gene from Modified Vaccinia Virus Ankara increases immunogenicity and isotype switching. Vaccine 29: 3276–3283. 21352940 10.1016/j.vaccine.2011.02.023 PMC3078999

[29] Talloczy Z, Jiang W, Virgin HW, Leib DA, Scheuner D, et al (2002) Regulation of starvation- and virus-induced autophagy by the eIF2alpha kinase signaling pathway. Proc Natl Acad Sci U S A 99: 190–195. 11756670 10.1073/pnas.012485299 PMC117537

[30] Lu B, Nakamura T, Inouye K, Li J, Tang Y, et al (2012) Novel role of PKR in inflammasome activation and HMGB1 release. Nature 488: 670–674 nature11290. 22801494 10.1038/nature11290 PMC4163918

[31] Hett EC, Slater LH, Mark KG, Kawate T, Monks BG, et al (2013) Chemical genetics reveals a kinase-independent role for protein kinase R in pyroptosis. Nat Chem Biol 9: 398–405 nchembio.1236 [pii];10.1038/nchembio.1236 [doi]. 23603659 10.1038/nchembio.1236 PMC6615456

[32] Child SJ, Hakki M, De Niro KL, Geballe AP (2004) Evasion of cellular antiviral responses by human cytomegalovirus TRS1 and IRS1. J Virol 78: 197–205. 14671101 10.1128/JVI.78.1.197-205.2004 PMC303427

[33] Tartaglia J, Perkus ME, Taylor J, Norton EK, Audonnet JC, et al (1992) NYVAC: a highly attenuated strain of vaccinia virus. Virology 188: 217–232. 1566575 10.1016/0042-6822(92)90752-b

[34] Beattie E, Paoletti E, Tartaglia J (1995) Distinct patterns of IFN sensitivity observed in cells infected with vaccinia K3L- and E3L- mutant viruses. Virology 210: 254–263. 7542414 10.1006/viro.1995.1342

[35] Beattie E, Tartaglia J, Paoletti E (1991) Vaccinia virus-encoded eIF-2 alpha homolog abrogates the antiviral effect of interferon. Virology 183: 419–422. 1711259 10.1016/0042-6822(91)90158-8

[36] Rahman MM, Liu J, Chan WM, Rothenburg S, McFadden G (2013) Myxoma Virus Protein M029 Is a Dual Function Immunomodulator that Inhibits PKR and Also Conscripts RHA/DHX9 to Promote Expanded Host Tropism and Viral Replication. PLoS Pathog 9: e1003465. 23853588 10.1371/journal.ppat.1003465 PMC3701710

[37] Marshall EE, Bierle CJ, Brune W, Geballe AP (2009) Essential role for either TRS1 or IRS1 in human cytomegalovirus replication. J Virol 83: 4112–4120. 19211736 10.1128/JVI.02489-08 PMC2668495

[38] Chenna R, Sugawara H, Koike T, Lopez R, Gibson TJ, et al (2003) Multiple sequence alignment with the Clustal series of programs. Nucleic Acids Res 31: 3497–3500. 12824352 10.1093/nar/gkg500 PMC168907

[39] Esposito J, Condit R, Obijeski J (1981) The preparation of orthopoxvirus DNA. J Virol Methods 2: 175–179. 6268651 10.1016/0166-0934(81)90036-7

[40] Simpson JT, Wong K, Jackman SD, Schein JE, Jones SJ, et al (2009) ABySS: a parallel assembler for short read sequence data. Genome Res 19: 1117–1123. 19251739 10.1101/gr.089532.108 PMC2694472

[41] Li H, Durbin R (2009) Fast and accurate short read alignment with Burrows-Wheeler transform. Bioinformatics 25: 1754–1760 btp324. 19451168 10.1093/bioinformatics/btp324 PMC2705234

[42] McKenna A, Hanna M, Banks E, Sivachenko A, Cibulskis K, et al (2010) The Genome Analysis Toolkit: a MapReduce framework for analyzing next-generation DNA sequencing data. Genome Res 20: 1297–1303. 20644199 10.1101/gr.107524.110 PMC2928508

[43] Li H, Durbin R (2010) Fast and accurate long-read alignment with Burrows-Wheeler transform. Bioinformatics 26: 589–595. 20080505 10.1093/bioinformatics/btp698 PMC2828108

[44] Kurtz S, Phillippy A, Delcher AL, Smoot M, Shumway M, et al (2004) Versatile and open software for comparing large genomes. Genome Biol 5: R12 10.1186/gb-2004-5-2-r12 [doi];gb-2004-5-2-r12 [pii]. 14759262 10.1186/gb-2004-5-2-r12 PMC395750

