# Supplementary figures and images for: Adaptive Gene Amplification As an Intermediate Step in the Expansion of Virus Host Range
Source: PLoS Pathog. 2014 Mar 13;10(3):e1004002. doi: 10.1371/journal.ppat.1004002 (PMC3953438; doi:10.1371/journal.ppat.1004002)

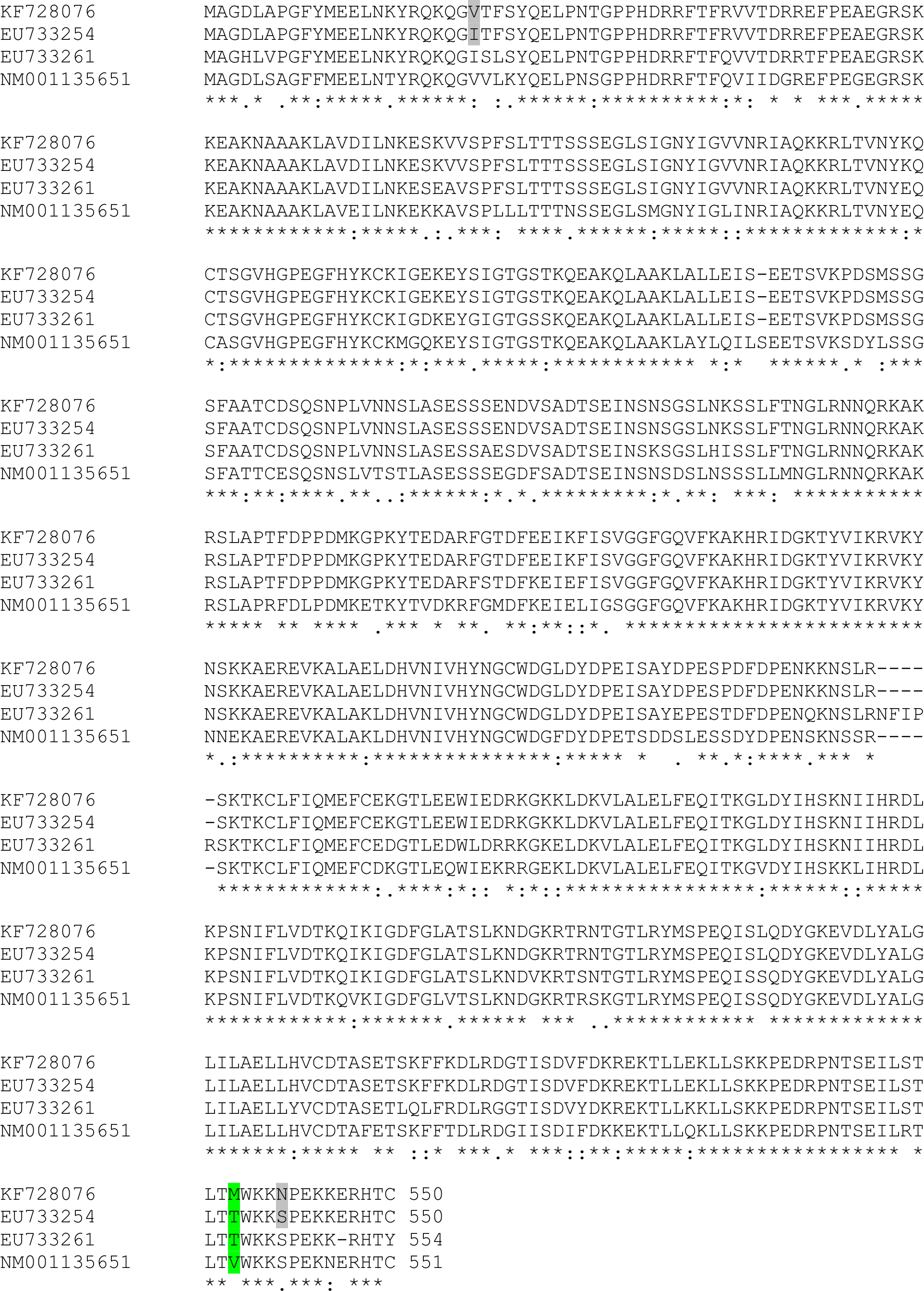

Supplement: Figure S2 — Amino acid alignment of African green monkey, human, and rhesus PKR. Predicted PKR amino acid sequences from AGM (PRO1190T1610 and EU733254), rhesus macaque (GenBank# EU733261), and human (GenBank # NM001135651) were aligned using CLUSTAL W (1.83) [38]. We identified three non-synonymous amino acid differences (grey or green highlighted residues) between the two AGM alleles. One of these differences is at a site that is evolving under positive selection in primates (green highlighted residue) [12]. Relative to PRO1190 PKR, rhesus and human PKR are 90.8% and 83.2% identical at the amino acid level, respectively. (TIF) [file ppat.1004002.s002.tif]

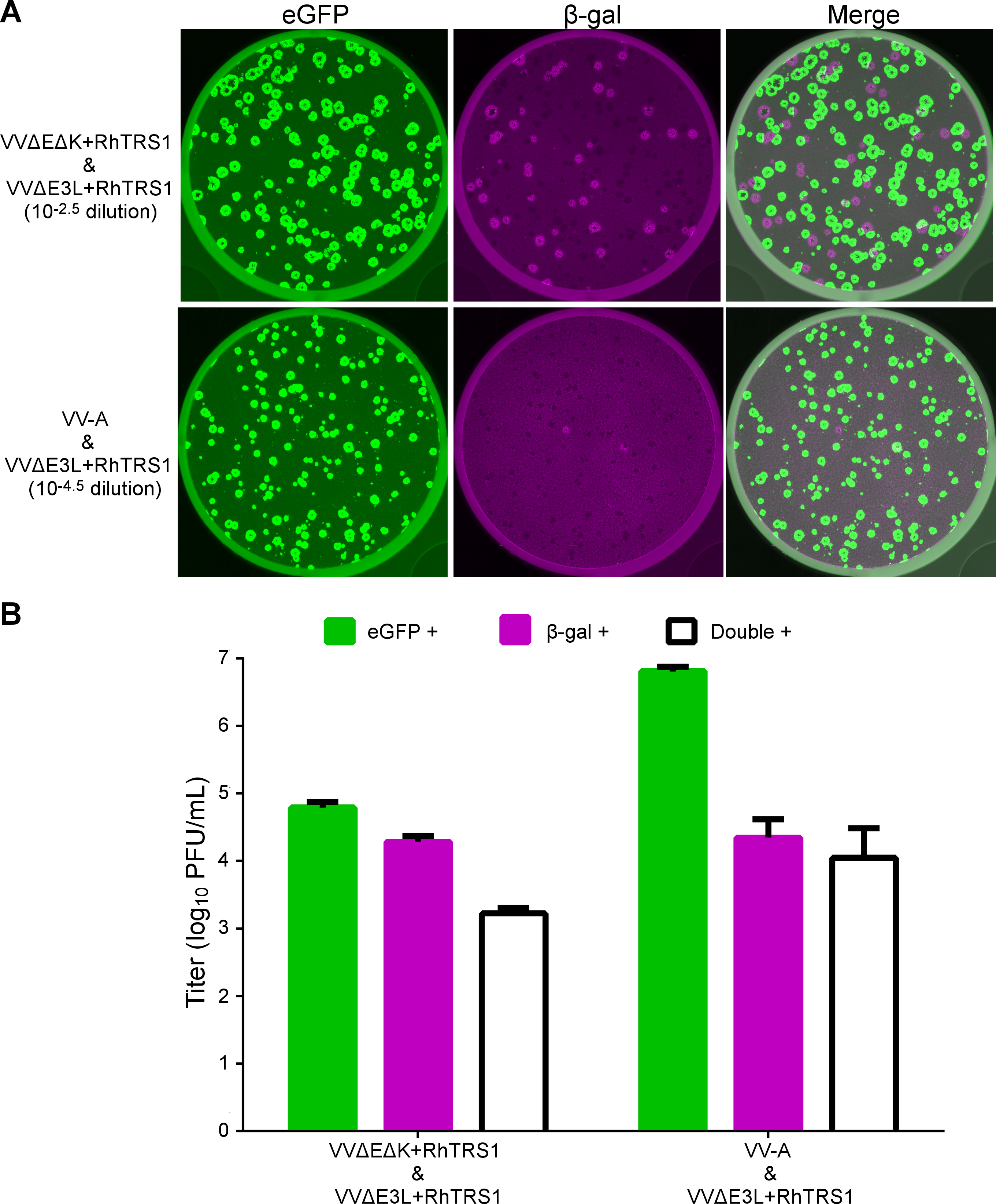

Supplement: Figure S3 — Increased fitness of VV-A compared to VVΔEΔK+RhTRS1 assessed by indirect competition assay. PRO1190 cells were co-infected with either VVΔEΔK+RhTRS1 or VV-A (MOI = 0.1) and the same competitor virus, VVΔE3L+RhTRS1 (MOI = 0.1). Two days post-infection viral progeny were collected and titered on BSC40 cells. (A) Representative Typhoon image of virus titer plates produced by infection with VVΔE+RhTRS1 in combination with either VVΔEΔK+RhTRS1 (top panels) or VV-A (bottom panels). (B) Plaques were scored for eGFP expression (VVΔEΔK+RhTRS1 or VV-A), β-gal expression (VVΔE3L+RhTRS1) or both (double +) as described in Materials and Methods. VV-A replicated ∼100-fold better than VVΔEΔK+RhTRS1 relative to VVΔE3L+RhTRS1. Data are represented as the mean +1 SD. (TIF) [file ppat.1004002.s003.tif]

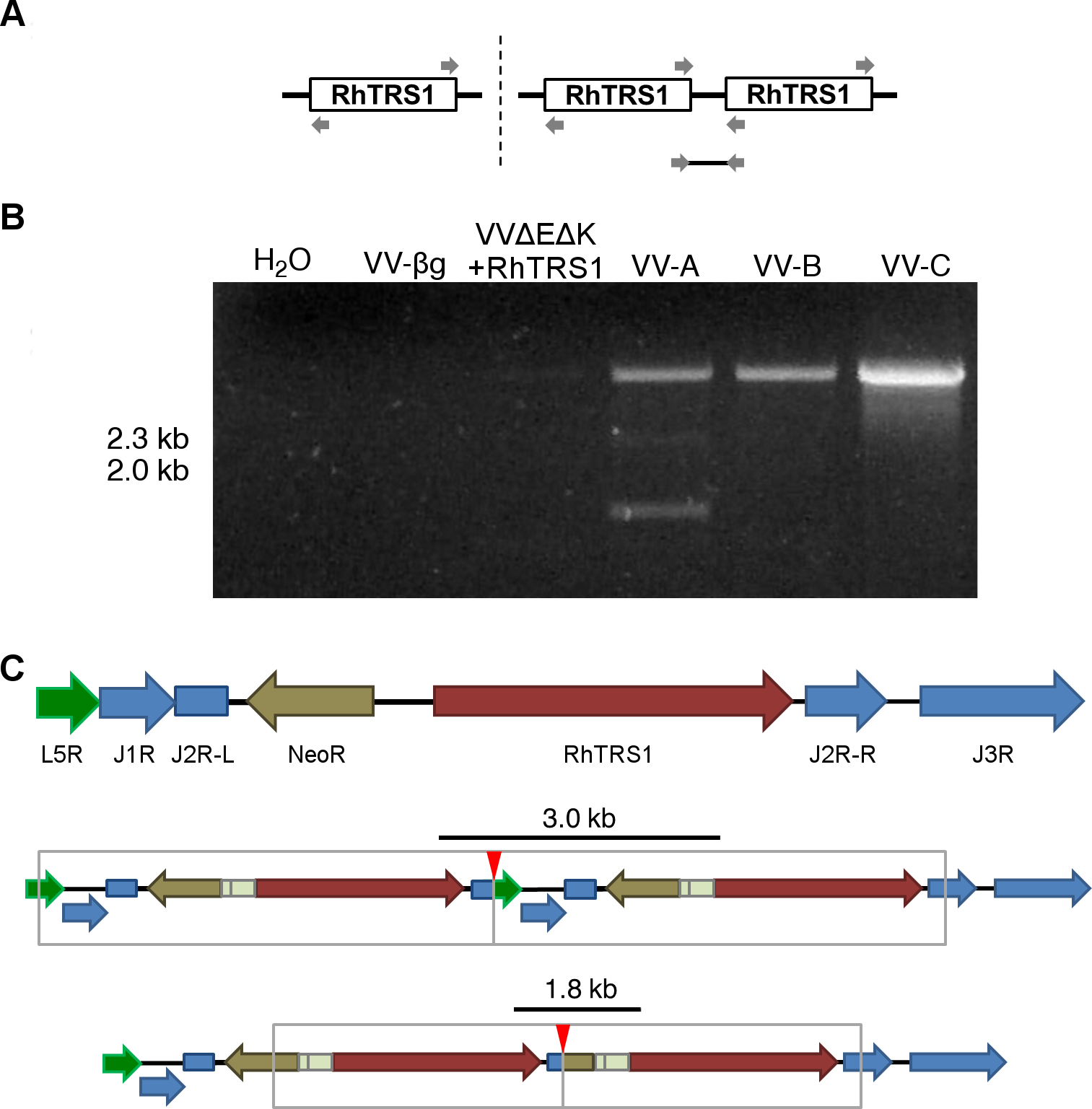

Supplement: Figure S4 — Predominant recombination sites identified in rhtrs1 locus amplification. (A) Schematic of externally directed PCR. Externally directed oligonucleotides (grey arrows) were designed to bind to the ends of rhtrs1 (white). Only tandem duplications will produce an amplification product. (B) Externally directed PCR revealed enrichment of rhtrs1 amplification products in the passaged virus pools. (C) Schematic of the initial rhtrs1 locus in VVΔEΔK+RhTRS1 (top), and the predominant recombination sites identified in all three passaged viruses (middle), or in VV-A alone (bottom). Vaccinia virus genes are colored green or blue, and exogenous sequences are colored dark red (rhtrs1) or brown (neoR). PCR products amplified by externally directed primers (black bars), the duplicated sequence (grey boxes), and the recombination sites (red arrowheads), are indicated. Recombination sites occur (middle) after nt 84267 (J2R) and before nt 83200 (L5R) relative to the reference vaccinia virus. Copenhagen strain (Genbank #M35027.1), or (bottom) after nt 84237 (J2R) and before nt 622 (neoR - pcDNA3.1, Invitrogen). (TIF) [file ppat.1004002.s004.tif]
