# Supplementary material for: Adaptive Gene Amplification As an Intermediate Step in the Expansion of Virus Host Range
Source: PLoS Pathog. 2014 Mar 13;10(3):e1004002. doi: 10.1371/journal.ppat.1004002 (PMC3953438; doi:10.1371/journal.ppat.1004002)
Supplement: Table S1 — Genomic coordinates for regions in whole genome alignment between Vaccinia copenhagen reference (GenBank M35027.1) and parental genome (VVΔEΔK+RhTRS1 genome) sequenced here, as determined by MUMmer 3.2.1 [44] . (DOCX) [file ppat.1004002.s005.docx]

**Table S1. Genomic coordinates for regions in whole genome alignment between Vaccinia copenhagen reference (GenBank M35027.1) and parental genome (VVΔEΔK+RhTRS1 genome) sequenced here, as determined by MUMmer 3.2.1** [44]

| Copenhagen reference genome coordinates | | | VVΔEΔK+RhTRS1 genome coordinates | | |
| --- | --- | --- | --- | --- | --- |
| Start | End | Notes | Start | End | Notes |
| 1 | 30365 |  | 1 | 30365 |  |
| 30363 | 30629 | K3L (deleted in VVΔEΔK+RhTRS1) | NA | NA |  |
| NA | NA |  | 30751 | 31209 | *E. coli* hypoxanthine-guanine phosphoribosyl transferase [35] |
| 30623 | 50910 |  | 31367 | 51678 |  |
| 50911 | 51483 | E3L (deleted in VVΔEΔK+RhTRS1) | NA | NA |  |
| NA | NA |  | 51700 | 52545 | eGFP |
| 51484 | 84100 |  | 52569 | 85185 |  |
| 83855 | 84130 | 5' J2R | 85186 | 85456 | 5' J2R |
| NA | NA |  | 85457 | 86251 | NeoR |
| NA | NA |  | 86662 | 88917 | rhTRS1_bioHis |
| 84131 | 191737 |  | 88922 | 196531 |  |
