# Supplementary material for: Adaptive Gene Amplification As an Intermediate Step in the Expansion of Virus Host Range
Source: PLoS Pathog. 2014 Mar 13;10(3):e1004002. doi: 10.1371/journal.ppat.1004002 (PMC3953438; doi:10.1371/journal.ppat.1004002)
Supplement: Table S2 — Substitution and short indel variants (<10 bp) present in parental genome (VVΔEΔK+RhTRS1) as compared to Vaccinia Copenhagen genome (excluding any in the inverted terminal repeat regions). Predicted effects upon protein coding genes as annotated in the Copenhagen reference are listed, with more than one line per variant indicating effects upon multiple overlapping gene models. (DOCX) [file ppat.1004002.s006.docx]

**Table S2. Substitution and short indel variants (<10 bp) present in parental genome (VVΔEΔK+RhTRS1) as compared to Vaccinia Copenhagen genome (excluding any in the inverted terminal repeat regions). Predicted effects upon protein coding genes as annotated in the Copenhagen reference are listed, with more than one line per variant indicating effects upon multiple overlapping gene models.**

| Position, Copenhagen genome | Copenhagen allele | Change in VVΔEΔK | Gene_name | Effect | Old AA / new AA | Old codon / new codon |
| --- | --- | --- | --- | --- | --- | --- |
| 23443 | C | T | C2L | SYNONYMOUS_CODING | R/R | agG/agA |
| 24256 | G | C | C1L | SYNONYMOUS_CODING | R/R | cgC/cgG |
| 25525 | G | C | N2L | NON_SYNONYMOUS_CODING | P/R | cCg/cGg |
| 30628 | A | T | K3L | START_LOST | M/K | aTg/aAg |
| 35080 | CG | GC | F3L | NON_SYNONYMOUS_CODING | T/S | acg/aGC |
| 35080 | CG | GC | F_ORF_A | NON_SYNONYMOUS_CODING | PV/PL | cccgta/ccGCta |
| 44312 | A | G | F13L | SYNONYMOUS_CODING | D/D | gaT/gaC |
| 46742 | CG | GC | F16L | NON_SYNONYMOUS_CODING | R/A | cga/GCa |
| 77258 | A | T | G7L | NON_SYNONYMOUS_CODING | V/E | gTg/gAg |
| 81834 | G | A | L3L | NON_SYNONYMOUS_CODING | L/F | Ctt/Ttt |
| 84328 | A | G | J2R | SYNONYMOUS_CODING | E/E | gaA/gaG |
| 85139 | T | C | J3R | NON_SYNONYMOUS_CODING | V/A | gTt/gCt |
| 104656 | C | T | D6R | NON_SYNONYMOUS_CODING | T/M | aCg/aTg |
| 148904 | A | T | A41L | SYNONYMOUS_CODING | T/T | acT/acA |
| 152700 | * | +C | A46R | FRAME_SHIFT | NA | NA |
| 152700 | * | +C | A_ORF_Q | FRAME_SHIFT | NA | NA |
