# Supplementary material for: Adaptive Gene Amplification As an Intermediate Step in the Expansion of Virus Host Range
Source: PLoS Pathog. 2014 Mar 13;10(3):e1004002. doi: 10.1371/journal.ppat.1004002 (PMC3953438; doi:10.1371/journal.ppat.1004002)
Supplement: Table S3 — Mutations identified in VV-A, VV-B and VV-C after four passages through HFF relative to vaccinia virus (strain Copenhagen). (DOCX) [file ppat.1004002.s007.docx]

**Table S3. Mutations identified in VV-A, VV-B and VV-C after four passages through HFF relative to vaccinia virus (strain Copenhagen).**

|  | | | | | VV-A HFF | VV-B HFF | VV-C HFF |
| --- | --- | --- | --- | --- | --- | --- | --- |
| Position | Reference Base(s) | Variant Base(s) | Gene | Effect | Allele Frequency | Allele Frequency | Allele Frequency |
| 39202 | T | C | F7L | mis:K43E:aag>Gag | 17.6% | 16.9% | 13.2% |
| 39203 | A | C | F7L | mis:N33K:aat>aaG | 20.8% | 21.2% | 13.2% |
| 42543 | A | AGT | F11L | Indel | 24.3% | 0.0% | 0.0% |
| 86598 | C | T | N/A* | N/A* | 7.6% | 3.5% | 5.2% |
| 89059 | GT | G | N/A* | N/A* | 0.6% | 7.7% | 3.5% |
| 99216 | A | ACAT | H4L | Indel:134DDVR>DDDVR | 14.4% | 0.0% | 5.5% |
| 142469 | C | T | A24R | mis:T1121M:acg>aTg | 47.0% | 0.0% | 0.0% |
| 146037 | T | TAA | A31R | indel | 46.1% | 0.0% | 0.0% |
| 148449 | TA | T | A35R | indel | 48.7% | 0.0% | 0.0% |
| 148499 | CA | C | A35R | indel | 29.8% | 0.0% | 3.6% |
| 148601 | CTA | C | A35R | indel | 0.0% | 0.0% | 13.0% |
| 148625 | TG | T | A35R | indel | 1.7% | 18.0% | 31.3% |
| 149912 | G | A | A37R | mis:S116N:agc>aAc | 0.0% | 64.6% | 6.9% |
| 150258 | C | CA | A37R | indel | 0.0% | 1.5% | 22.0% |
| * Not annotated |  |  |  |  |  |  |  |
