# Supplementary material for: Adaptive Gene Amplification As an Intermediate Step in the Expansion of Virus Host Range
Source: PLoS Pathog. 2014 Mar 13;10(3):e1004002. doi: 10.1371/journal.ppat.1004002 (PMC3953438; doi:10.1371/journal.ppat.1004002)
Supplement: Table S4 — Accession numbers. (DOCX) [file ppat.1004002.s008.docx]

**Table S4. Accession numbers.**

| **Sequence name** | **Accession number** |
| --- | --- |
| AGM PKR | EU733254 |
| PRO1190 PKR | KF728076-7 |
| Rhesus macaque PKR | EU733261 |
| Human PKR | NM001135651 |
| Vaccinia virus Copenhagen | M35027.1 |
| Vaccinia virus deep sequencing short read data | SRP033208 |
